# Supplementary material for: Experimental evidence that chronic outgroup conflict reduces reproductive success in a cooperatively breeding fish
Source: eLife. 2022 Sep 14;11:e72567. doi: 10.7554/eLife.72567 (PMC9473690; doi:10.7554/eLife.72567)
Supplement: Supplementary file 1. — Effect of outgroup conflict on (a) latency to spawn (days) and (b) inter-clutch interval (days); latency to spawn was log10-transformed. Female size relates to dominant female standard length at the start of the study. Tank-triplet and/or group identity nested within tank-triplet were fitted as random intercepts (with variances shown). The reference level for Treatment was Control. Each table section displays the final model, with removed non-significant interactions below. [file elife-72567-supp1.docx]

**Supplementary File 1.** **Statistical summary of linear mixed models testing the effect of chronic outgroup conflict (Intruded vs Control, Experiment I) on breeding timings.** Effect of outgroup conflict on (a) latency to spawn (days), and (b) inter-clutch interval (days); latency to spawn was log_10_-transformed. Female size relates to dominant female standard length at the start of the study. Tank-triplet and/or group identity nested within tank-triplet were fitted as random intercepts (with variances shown). The reference level for Treatment was Control. Each table section displays the final model, with removed non-significant interactions below.

| **a. Latency to spawn (N = 13 groups)** | | | | | | |
| --- | --- | --- | --- | --- | --- | --- |
| Random terms: Tank-triplet: 0.13; Residual: 0.06 | | | | | | |
| FINAL MODEL | estimate ± s.e. | C.I. | d.f. | t-value | p | *Χ*^2^ |
| Intercept | 3.23 ± 1.43 | 0.49 – 5.92 | 7.63 | 2.26 | 0.055 |  |
| Treatment |  |  | 1 |  | 0.312 | 1.02 |
| Treatment (Intruded) | -0.15 ± 0.16 | -0.46 – 0.18 | 5.02 | -0.95 | 0.388 |  |
| Female size | -0.04 ± 0.03 | -0.09 – 0.01 | 7.78 | -1.48 | 0.177 |  |
| REMOVED INTERACTION |  |  | d.f. |  | p | Χ^2^ |
| Treatment x Female size |  |  | 1 |  | 0.493 | 0.47 |
| **b. Inter-clutch interval (N = 21 intervals)** | | | | | | |
| Random terms: Tank-triplet: 36.16; Tank-triplet/Group: 47.23; Residual: 4.66 | | | | | | |
| FINAL MODEL | estimate ± s.e. | C.I. | d.f. | t-value | p | Χ^2^ |
| Intercept | -5.72 ± 9.85 | -26.08 – 13.63 | 10.82 | -0.58 | 0.570 |  |
| Treatment |  |  | 1 |  | 0.049 | 3.89 |
| Treatment (Intruded) | 8.88 ± 4.34 | 0.06 – 17.71 | 5.86 | 2.05 | 0.088 |  |
| Female size | 0.49 ± 0.17 | 0.16 – 0.85 | 8.64 | 2.97 | 0.017 |  |
| REMOVED INTERACTION |  |  | d.f. |  | p | Χ^2^ |
| Treatment x Female size |  |  | 1 |  | 0.373 | 0.80 |
