# Supplementary material for: Experimental evidence that chronic outgroup conflict reduces reproductive success in a cooperatively breeding fish
Source: eLife. 2022 Sep 14;11:e72567. doi: 10.7554/eLife.72567 (PMC9473690; doi:10.7554/eLife.72567)
Supplement: Supplementary file 2. — Effect of outgroup conflict on clutch when females could spawn repeatedly throughout the study (Experiment I). Female size relates to dominant female standard length measurement made closest in time to the production of each clutch (start or end of study). Tank-triplet and group identity nested within tank-triplet were fitted as random intercepts (with variances shown). The reference level for Treatment was Control. Table displays the final model, with removed non-significant interactions below. [file elife-72567-supp2.docx]

**Supplementary File 2. Statistical summary of linear mixed models testing the effect of chronic outgroup conflict (Intruded vs Control) on clutch size.** Effect of outgroup conflict on clutch when females could spawn repeatedly throughout the study (Experiment I). Female size relates to dominant female standard length measurement made closest in time to the production of each clutch (start or end of study). Tank-triplet and group identity nested within tank-triplet were fitted as random intercepts (with variances shown). The reference level for Treatment was Control. Table displays the final model, with removed non-significant interactions below.

| **Clutch size (N=34 clutches)** | | | | | | |
| --- | --- | --- | --- | --- | --- | --- |
| Random terms: Tank-triplet: 0.0; Tank-triplet/Group: 1692.9; Residual: 739.2 | | | | | | |
| FINAL MODEL | estimate ± s.e. | C.I. | d.f. | t-value | p | Χ^2^ |
| Intercept | -113.98 ± 93.09 | -291.06 – 60.54 | 29.07 | -1.22 | 0.231 |  |
| Treatment |  |  | 1 |  | 0.091 | 2.85 |
| Treatment (Intruded) | 39.93 ± 24.95 | -7.29 – 87.63 | 10.44 | 1.60 | 0.139 |  |
| Treatment duration | 0.69 ± 0.27 | 0.12 – 1.19 | 25.46 | 2.58 | 0.016 |  |
| Female size | 3.07 ± 1.77 | -0.24 – 6.45 | 29.23 | 1.74 | 0.093 |  |
| REMOVED INTERACTIONS |  |  | d.f. |  | p | Χ^2^ |
| Treatment x Female size |  |  | 1 |  | 0.615 | 0.25 |
| Treatment x Treatment duration |  |  | 1 |  | 0.059 | 3.55 |
