# Supplementary material for: Experimental evidence that chronic outgroup conflict reduces reproductive success in a cooperatively breeding fish
Source: eLife. 2022 Sep 14;11:e72567. doi: 10.7554/eLife.72567 (PMC9473690; doi:10.7554/eLife.72567)
Supplement: Supplementary file 3. — Effect of outgroup conflict on egg (a) volume (mm3), (b) dry weight (mg), (c) lipid content (µg), and (d) protein content (µg). Female size relates to dominant female standard length at the start of the study. Tank-triplet and group identity nested within tank-triplet were fitted as random intercepts (with variances shown). The reference level for Treatment was Control. Each table section displays the final model, with removed non-significant interactions below. For fixed effects included in significant interactions, only parameter estimates are shown. [file elife-72567-supp3.docx]

**Supplementary File 3.** **Statistical summary of linear mixed models testing the effect of chronic outgroup conflict (Intruded vs Control, Experiment II) on mean morphological and physiological egg characters.** Effect of outgroup conflict on egg (a) volume (mm^3^), (b) dry weight (mg), (c) lipid content (µg) and (d) protein content (µg). Female size relates to dominant female standard length at the start of the study. Tank-triplet and group identity nested within tank-triplet were fitted as random intercepts (with variances shown). The reference level for Treatment was Control. Each table section displays the final model, with removed non-significant interactions below. For fixed effects included in significant interactions, only parameter estimates are shown.

| **a. Egg dry weight (N=15 clutches)** | | | | | | |
| --- | --- | --- | --- | --- | --- | --- |
| Random terms: Tank-triplet: 0.000; Tank-triplet/Group: 0.003; Residual: 0.001 | | | | | | |
| FINAL MODEL | estimate ± s.e. | C.I. | df | t-value | p | Χ^2^ |
| Intercept | 0.327 ± 0.189 | 0.004 – 0.651 | 10.01 | 1.73 | 0.115 |  |
| Treatment |  |  | 1 |  | 0.356 | 0.85 |
| Treatment (Intruded) | -0.026 ± 0.035 | -0.085 – 0.033 | 9.95 | -0.75 | 0.468 |  |
| Treatment duration | 0.001 ± 0.000 | -0.001 – 0.003 | 10.12 | 0.73 | 0.484 |  |
| Clutch size | 0.000 ± 0.000 | -0.000 – 0.000 | 10.07 | 0.51 | 0.623 |  |
| Female size | 0.001 ± 0.003 | -0.005 – 0.007 | 10.03 | 0.32 | 0.757 |  |
| REMOVED INTERACTIONS |  |  | d.f. |  | p | *Χ*^2^ |
| Treatment x Female size |  |  | 1 |  | 0.698 | 0.15 |
| Treatment x Treatment duration | |  | 1 |  | 0.254 | 1.30 |
| **b. Egg volume (N=15 clutches)** | | | | | | |
| Random terms: Tank-triplet: 0.00; Tank-triplet/Group: 0.27; Residual: 0.57 | | | | | | |
| FINAL MODEL | estimate ± s.e. | C.I. | df | t-value | p | Χ^2^ |
| Intercept | -0.402 ± 1.863 | -3.436 — 2.647 | 9.25 | -0.22 | 0.834 |  |
| Treatment (Intruded) | 1.012 ± 0.797 |  |  |  |  |  |
| Treatment duration | 0.050 ± 0.021 |  |  |  |  |  |
| Clutch size | 0.001 ± 0.003 | -0.004 — 0.006 | *9.36* | 0.29 | 0.780 |  |
| Female size | 0.018 ± 0.031 | -0.033 — 0.069 | 9.28 | 0.58 | 0.575 |  |
| Treatment x Treatment duration | |  | 1 |  | 0.037 | 4.34 |
| Intruded x Treatment duration | -0.043 ± 0.025 | -0.083 — -0.003 | 9.51 | -1.76 | 0.111 |  |
| REMOVED INTERACTION |  |  | d.f. |  | p | *Χ*^2^ |
| Treatment x Female size |  |  | 1 |  | 0.332 | 0.94 |
| **c. Egg lipid content (N=15 clutches)** | | | | | | |
| Random terms: Tank-triplet: 0.00; Tank-triplet/Group: 0.96; Residual: 0.71 | | | | | | |
| FINAL MODEL | estimate ± s.e. | C.I. | df | t-value | p | Χ^2^ |
| Intercept | 7.48 ± 3.67 | 1.21 — 13.77 | 10.08 | 2.04 | 0.070 |  |
| Treatment |  |  | 1 |  | 0.797 | 0.07 |
| Treatment (Intruded) | -0.13 ± 0.67 | -1.27 — 1.01 | 9.91 | -0.19 | 0.853 |  |
| Treatment duration | 0.03 ± 0.03 | -0.02 — 0.07 | 10.13 | 0.99 | 0.347 |  |
| Clutch size | 0.00 ± 0.01 | -0.01 — 0.01 | 10.16 | 0.73 | 0.484 |  |
| Female size | -0.03 ± 0.06 | -0.14 — 0.08 | 10.11 | -0.52 | 0.617 |  |
| REMOVED INTERACTIONS |  |  | d.f. |  | p | *Χ*^2^ |
| Treatment x Female size |  |  | 1 |  | 0.735 | 0.11 |
| Treatment x Treatment duration | |  | 1 |  | 0.290 | 1.12 |
| **d. Egg protein content (N=15 clutches)** | | | | | | |
| Random terms: Tank-triplet: 207.67; Tank-triplet/Group: 0.00; Residual: 10.72 | | | | | | |
| FINAL MODEL | estimate ± s.e. | C.I. | df | t-value | p | Χ^2^ |
| Intercept | 61.84 ± 38.39 | -9.50 — 130.98 | 9.7 | 1.61 | 0.139 |  |
| Treatment (Intruded) | 175.74 ± 29.27 |  |  |  |  |  |
| Treatment duration | -0.18 ± 0.29 |  |  |  |  |  |
| Clutch size | 0.18 ± 0.05 | 0.10 — 0.26 | 15.36 | 4.04 | 0.001 |  |
| Female size | 0.59 ± 0.60 |  |  |  |  |  |
| Treatment x Treatment duration | |  | 1 |  | <0.001 | 18.50 |
| Intruded x Treatment duration | -1.66 ± 0.30 | -2.19 — -1.13 | 15.98 | -5.55 | <0.001 |  |
| Treatment x Female size |  |  | 1 |  | 0.007 | 10.09 |
| Intruded x Female size | -1.88 ± 0.40 | -2.59 — -1.13 | 22.68 | -4.70 | <0.001 |  |
