# Supplementary material for: Experimental evidence that chronic outgroup conflict reduces reproductive success in a cooperatively breeding fish
Source: eLife. 2022 Sep 14;11:e72567. doi: 10.7554/eLife.72567 (PMC9473690; doi:10.7554/eLife.72567)
Supplement: Supplementary file 4. — Effect of outgroup conflict on (a) clutch visits and (b) caring (egg-cleaning and fanning) events; both analysed using negative binomial GLMMs with a ‘log’ link function. Tank-triplet and group identity nested within tank-triplet were fitted as random intercepts (with variances shown). The reference level for Treatment was Control. Each table section displays the final model, with removed non-significant interactions below. For fixed effects included in significant interactions, only parameter estimates are shown. [file elife-72567-supp4.docx]

**Supplementary File 4.** **Statistical summary of generalised linear mixed models (GLMMs) testing the effect of chronic outgroup conflict (Intruded vs Control, Experiment I) on number of parental-care behaviours performed during a 10-min period.** Effect of outgroup conflict on (a) clutch visits and (b) caring (egg-cleaning and fanning) events; both analysed using negative binomial GLMMs with a “log” link function. Tank-triplet and group identity nested within tank-triplet were fitted as random intercepts (with variances shown). The reference level for Treatment was Control. Each table section displays the final model, with removed non-significant interactions below. For fixed effects included in significant interactions, only parameter estimates are shown.

| **a. Number of clutch visits (N=33 clutches)** | | | | | | |
| --- | --- | --- | --- | --- | --- | --- |
| Random terms: Tank-triplet: 0.00; Tank-triplet/Group: 0.02 | | | | | | |
| FINAL MODEL | estimate ± s.e. | C.I. | d.f. | Z value | p | Χ^2^ |
| Intercept | 3.285 ± 0.208 | 2.847 – 3.689 |  | 15.77 | <0.001 |  |
| Treatment (Intruded) | -0.415 ± 0.263 |  |  |  |  |  |
| Treatment duration | -0.001 ± 0.003 |  |  |  |  |  |
| Clutch size | -0.000 ± 0.002 | -0.003 – 0.003 |  | -0.052 | 0.958 |  |
| Treatment x Treatment duration |  |  | 1 |  | 0.023 | 5.17 |
| Intruded x Treatment duration | 0.013 ± 0.006 | 0.002 – 0.024 |  | 2.34 | 0.020 |  |
| REMOVED INTERACTION |  |  | d.f. |  | p | Χ^2^ |
| Treatment x Clutch size |  |  | 1 |  | 0.958 | <0.01 |
| **b. Number of caring events (N=33 clutches)** | | | | | | |
| Random terms: Tank-triplet: 0.00; Tank-triplet/Group: 0.06 | | | | | | |
| FINAL MODEL | estimate ± s.e. | C.I. | d.f. | Z value | p | Χ^2^ |
| Intercept | 3.004 ± 0.260 | 2.494 – 3.514 |  | 11.54 | <0.001 |  |
| Treatment (Intruded) | -0.685 ± 0.341 |  |  |  |  |  |
| Treatment duration | 0.003 ± 0.004 |  |  |  |  |  |
| Clutch size | 0.001 ± 0.002 | -0.003 – 0.005 |  | 0.51 | 0.612 |  |
| Treatment x Treatment duration |  |  | 1 |  | 0.033 | 4.54 |
| Intruded x Treatment duration | 0.015 ± 0.007 | 0.001 – 0.028 |  | 2.17 | 0.030 |  |
| REMOVED INTERACTION |  |  | d.f. |  | p | *Χ*^2^ |
| Treatment x Clutch size |  |  | 1 |  | 0.866 | 0.03 |
