# Supplementary material for: Experimental evidence that chronic outgroup conflict reduces reproductive success in a cooperatively breeding fish
Source: eLife. 2022 Sep 14;11:e72567. doi: 10.7554/eLife.72567 (PMC9473690; doi:10.7554/eLife.72567)
Supplement: Supplementary file 5. — Effect of outgroup conflict on time spent on (a) clutch visits (linear mixed model, LMM) and (b) caring (egg-cleaning and fanning) events (generalised linear mixed model [GLMM] with ‘gaussian’ family and ‘log’ link function). Tank-triplet and group identity nested within tank-triplet were fitted as random intercepts (with variances shown). The reference level for Treatment was Control. Each table section displays the final model, with removed non-significant interactions below. For fixed effects included in significant interactions, only parameter estimates are shown. [file elife-72567-supp5.docx]

**Supplementary File 5.** **Statistical summary of mixed models testing the effect of chronic outgroup conflict (Intruded vs Control, Experiment I) on time spent on parental-care behaviour during a 10-min period.** Effect of outgroup conflict on time spent on (a) clutch visits (linear mixed model, LMM) and (b) caring (egg-cleaning and fanning) events (generalized linear mixed model (GLMM) with ”gaussian” family and “log” link function). Tank-triplet and group identity nested within tank-triplet were fitted as random intercepts (with variances shown). The reference level for Treatment was Control. Each table section displays the final model, with removed non-significant interactions below. For fixed effects included in significant interactions, only parameter estimates are shown.

| **a. Time spent in the vicinity of clutches (N = 33 clutches)** | | | | | | |
| --- | --- | --- | --- | --- | --- | --- |
| Random terms: Tank-triplet: 521.4; Tank-triplet/Group: 0.0; Residual: 3019.2 | | | | | | |
| FINAL MODEL | estimate ± s.e. | C.I. | df | t-value | p | Χ^2^ |
| Intercept | 136.84 ± 32.87 | 74.40 – 197.78 | 27.39 | 4.16 | <0.001 |  |
| Treatment (Intruded) | -71.67 ± 41.90 |  |  |  |  |  |
| Treatment duration | 0.34 ± 0.56 |  |  |  |  |  |
| Clutch size | -0.11 ± 0.23 | -0.53 – 0.32 | 26.88 | -0.49 | 0.626 |  |
| Treatment x Treatment duration |  |  | 1 |  | 0.027 | 4.92 |
| Intruded x Treatment duration | 2.01 ± 0.88 | 0.25 – 3.64 | 24.97 | 2.29 | 0.031 |  |
| REMOVED INTERACTION |  |  | df |  | p | *Χ*^2^ |
| Treatment x Clutch size |  |  | 1 |  | 0.358 | 0.85 |
| **b. Time spent on parental-care activities (N = 33 clutches)** | | | | | | |
| Random terms: Tank-triplet: 0.0; Tank-triplet/Group: 104.1; Residual: 548.0 | | | | | | |
| FINAL MODEL | estimate ± s.e. | C.I. | df | t-value | p | *Χ*^2^ |
| Intercept | 3.846 ± 0.309 | 2.239 – 4.452 |  | 12.43 | <0.001 |  |
| Treatment (Intruded) | -1.175 ± 0.461 |  |  |  |  |  |
| Treatment duration | 0.004 ± 0.004 |  |  |  |  |  |
| Clutch size | 0.003 ± 0.003 | -0.003 – 0.010 |  | 1.01 | 0.314 |  |
| Treatment x Treatment duration |  |  | 1 |  | 0.004 | 8.29 |
| Intruded x Treatment duration | 0.021 ± 0.008 | -0.006 – 0.035 |  | 2.75 | 0.006 |  |
| REMOVED INTERACTIONS |  |  | df |  | p | *Χ*^2^ |
| Treatment x Clutch size |  |  | 1 |  | 0.714 | 0.13 |
