# Supplementary material for: Experimental evidence that chronic outgroup conflict reduces reproductive success in a cooperatively breeding fish
Source: eLife. 2022 Sep 14;11:e72567. doi: 10.7554/eLife.72567 (PMC9473690; doi:10.7554/eLife.72567)
Supplement: Supplementary file 6. — Tank-triplet and group identity nested within tank-triplet were fitted as random intercepts (with variances shown). The reference level for Treatment was Control. Table shows the final model with removed non-significant interactions below. [file elife-72567-supp6.docx]

**Supplementary File 6. Statistical summary of a linear mixed model testing the effect of chronic outgroup conflict (Intruded vs Control, Experiment I) on hatching success (%).** Tank-triplet and group identity nested within tank-triplet were fitted as random intercepts (with variances shown). The reference level for Treatment was Control. Table shows the final model with removed non-significant interactions below.

| **Hatching success (N=21 clutches)** | | | | | | |
| --- | --- | --- | --- | --- | --- | --- |
| Random terms: Tank-triplet: 154.8; Tank-triplet/Group: 488.0; Residual: 698.7 | | | | | | |
| FINAL MODEL | estimate ± s.e. | C.I. | df | t-value | p | *Χ*^2^ |
| Intercept | 57.71 ± 24.59 | 11.75 – 112.41 | 15.19 | 2.35 | 0.033 |  |
| Treatment |  |  | 1 |  | 0.782 | 0.08 |
| Treatment (Intruded) | 7.25 ± 19.69 | -33.12 – 43.47 | 3.33 | 0.37 | 0.735 |  |
| Treatment duration | -0.28 ± 0.33 | -1.00 – 0.32 | 15.11 | -0.85 | 0.411 |  |
| Clutch size | 0.01 ± 0.25 | -0.51 – 0.47 | 9.10 | 0.05 | 0.960 |  |
| REMOVED INTERACTIONS |  |  | df |  | p | *Χ*^2^ |
| Treatment x Treatment duration |  |  | 1 |  | 0.186 | 1.75 |
| Treatment x Clutch size |  |  | 1 |  | 0.111 | 2.55 |
