# Supplementary material for: Experimental evidence that chronic outgroup conflict reduces reproductive success in a cooperatively breeding fish
Source: eLife. 2022 Sep 14;11:e72567. doi: 10.7554/eLife.72567 (PMC9473690; doi:10.7554/eLife.72567)
Supplement: Supplementary file 7. — Tank-triplet and group identity nested within tank-triplet were fitted as random intercepts (with variances shown); clutch size at laying was included as an offset. The reference level for Treatment was Control. Table displays the final model. For fixed effects included in the significant interaction, only parameter estimates are shown. [file elife-72567-supp7.docx]

**Supplementary File 7.** **Statistical summary of a linear mixed model testing the effect of chronic outgroup conflict (Intruded vs Control, Experiment I) on offspring survival to 1-month post-hatching.** Tank-triplet and group identity nested within tank-triplet were fitted as random intercepts (with variances shown); clutch size at laying was included as an offset. The reference level for Treatment was Control. Table displays the final model. For fixed effects included in the significant interaction, only parameter estimates are shown.

| **Number of offspring surviving to 1-month of age (N=32 clutches)** | | | | | | |
| --- | --- | --- | --- | --- | --- | --- |
| Random terms: Tank-triplet: 0; Tank-triplet/Group: 1720; Residual: 476 | | | | | | |
| FINAL MODEL | estimate ± s.e. | C.I. | df | t-value | p | *Χ*^2^ |
| Intercept | -63.30 ± 26.30 | -114.84–-11.75 | 27.75 | -2.41 | 0.023 |  |
| Treatment (Intruded) | 90.61 ± 37.81 |  |  |  |  |  |
| Treatment duration | 0.01 ± 0.28 |  |  |  |  |  |
| Treatment x Treatment duration |  |  | 1 |  | <0.001 | 13.31 |
| Intruded x Treatment duration | -1.83 ± 0.42 | -2.64–-1.02 | 18.56 | -4.40 | <0.001 |  |
