# Supplementary material for: Experimental evidence that chronic outgroup conflict reduces reproductive success in a cooperatively breeding fish
Source: eLife. 2022 Sep 14;11:e72567. doi: 10.7554/eLife.72567 (PMC9473690; doi:10.7554/eLife.72567)
Supplement: Supplementary file 8. — Effect of outgroup conflict on (a) mean pre-stimulus activity (%), (b) mean pre-stimulus nearest-neighbour distance (cm), (c) mean latency to freeze (s) post-stimulus, and (d) latency (s) to become active post-stimulus. Tank-triplet and group identity nested within tank-triplet were fitted as random intercepts (with variances shown). The reference level for Treatment was Control. Each table section displays the final model, with removed non-significant interactions below. For fixed effects included in significant interactions, only parameter estimates are shown. [file elife-72567-supp8.docx]

**Supplementary File 8.** **Statistical summary of linear mixed models testing the effect of chronic outgroup conflict (Intruded vs Control, Experiment I) on offspring behaviour.** Effect of outgroup conflict on (a) mean pre-stimulus activity (%), (b) mean pre-stimulus nearest-neighbour distance (cm) (c) mean latency to freeze (s) post-stimulus, and (d) latency (s) to become active post-stimulus. Tank-triplet and group identity nested within tank-triplet were fitted as random intercepts (with variances shown). The reference level for Treatment was Control. Each table section displays the final model, with removed non-significant interactions below. For fixed effects included in significant interactions, only parameter estimates are shown.

| **a. Pre-stimulus activity (N=21 clutches)** | | | | | | |
| --- | --- | --- | --- | --- | --- | --- |
| Random terms: Tank-triplet: 0.02; Tank-triplet/Group: 0.00; Residual: 0.03 | | | | | | |
| FINAL MODEL | estimate ± s.e. | C.I. | df | t-value | p | *Χ*^2^ |
| Intercept | 0.913 ± 0.593 | -0.16 – 2.09 | 15.24 | 1.54 | 0.144 |  |
| Treatment |  |  | 1 |  | 0.194 | 1.69 |
| Treatment (Intruded) | 0.149 ± 0.0.117 | -0.09 – 0.37 | 15.83 | 1.26 | 0.224 |  |
| Treatment duration | -0.004 ± 0.002 | -0.01 – 0.00 | 13.93 | -2.21 | 0.044 |  |
| Offspring size | -0.020 ± 0.036 | -0.09 – 0.05 | 12.15 | -0.55 | 0.592 |  |
| Number of offspring in test | 0.029 ± 0.037 | -0.05 – 0.09 | 15.68 | 0.78 | 0.449 |  |
| REMOVED INTERACTION |  |  | df |  | p | *Χ*^2^ |
| Treatment x Treatment duration |  |  | 1 |  | 0.151 | 2.06 |
| **b. Nearest-neighbour distance (N=21 clutches)** | | | | | | |
| Random terms: Tank-triplet: 0.06; Tank-triplet/Group: 0.01; Residual: 0.34 | | | | | | |
| FINAL MODEL | estimate ± s.e. | C.I. | df | t-value | p | *Χ*^2^ |
| Intercept | 7.08 ± 1.90 | 3.30 – 10.76 | 15.67 | 3.73 | 0.002 |  |
| Treatment |  |  | 1 |  | 0.745 | 0.11 |
| Treatment (Intruded) | 0.11 ± 0.37 | -0.57 – 0.75 | 9.92 | 0.29 | 0.780 |  |
| Treatment duration | -0.01 ± 0.01 | -0.02 – 0.01 | 14.78 | -1.00 | 0.332 |  |
| Number of offspring in test | -0.44 ± 0.12 | -0.65 – -0.20 | 11.39 | -3.69 | 0.003 |  |
| Offspring size | 0.05 ± 0.12 | -0.17 – -0.26 | 11.42 | 0.40 | 0.697 |  |
| REMOVED INTERACTION |  |  | df |  | p | *Χ*^2^ |
| Treatment x Treatment duration |  |  | 1 |  | 0.100 | 2.70 |
| **c. Latency to freeze (N=21 clutches)** | | | | | | |
| Random terms: Tank-triplet: 0.93; Tank-triplet/Group: 0.00; Residual: 0.34 | | | | | | |
| FINAL MODEL | estimate ± s.e. | C.I. | df | t-value | p | *Χ*^2^ |
| Intercept | -4.15 ± 2.53 | -9.97–0.13 | 10.38 | -1.64 | 0.130 |  |
| Treatment |  |  | 1 |  | 0.806 | 0.06 |
| Treatment (Intruded) | 0.13 ± 0.53 | -0.76–1.02 | 12.77 | 0.24 | 0.815 |  |
| Treatment duration | 0.03 ± 0.01 | 0.02–0.06 | 9.41 | 3.18 | 0.011 |  |
| Number of offspring in test | 0.25 ± 0.13 | 0.02–0.47 | 7.48 | 1.86 | 0.102 |  |
| Offspring size | -0.15 ± 0.15 | -0.43–0.24 | 11.07 | -0.98 | 0.348 |  |
| Pre-stimulus activity levels | 4.89 ± 1.01 | 3.08–6.70 | 10.08 | 4.84 | <0.001 |  |
| REMOVED INTERACTION |  |  | df |  | p | *Χ*^2^ |
| Treatment x Treatment duration |  |  | 1 |  | 0.666 | 0.19 |
| **d. Latency to first movement post-stimulus (N=21 clutches)** | | | | | | |
| Random terms: Tank-triplet: 0.0; Tank-triplet/Group: 0.0; Residual: 135.3 | | | | | | |
| FINAL MODEL | estimate ± s.e. | C.I. | df | t-value | p | *Χ*^2^ |
| Intercept | 29.44 ± 38.52 | -34.79 – 94.02 | 14.00 | 0.76 | 0.457 |  |
| Treatment (Intruded) | 35.99 ± 17.49 |  |  |  |  |  |
| Treatment duration | 0.13 ± 0.21 |  |  |  |  |  |
| Number of offspring in test | -2.25 ± 2.27 | -6.06 – 1.55 | 14.00 | -0.990 | 0.337 |  |
| Offspring size | 3.15 ± 2.19 | -0.51 – 8.76 | 14.00 | 1.440 | 0.171 |  |
| Pre-stimulus activity levels | -30.43 ± 14.15 | -54.13 – -6.71 | 14.00 | -2.150 | 0.049 |  |
| Treatment x Treatment duration |  |  | 1 |  | 0.032 | 4.62 |
| Intruded x Treatment duration | -0.49 ± 0.26 | -0.93 – -0.04 | 14.00 | -1.860 | 0.085 |  |
