# Supplementary material for: Experimental evidence that chronic outgroup conflict reduces reproductive success in a cooperatively breeding fish
Source: eLife. 2022 Sep 14;11:e72567. doi: 10.7554/eLife.72567 (PMC9473690; doi:10.7554/eLife.72567)
Supplement: Supplementary file 9. — Effect of outgroup size on (a) mean standard length (mm) and (b) mean dry weight (mg). Tank-triplet and group identity nested within tank-triplet were fitted as random intercepts (with variances shown). The reference level for Treatment was Control. Each table section displays the final model, with removed non-significant interactions below. [file elife-72567-supp9.docx]

**Supplementary File 9.** **Statistical summary of linear mixed models testing the effect of chronic outgroup conflict (Intruded vs Control, Experiment I) on offspring size.** Effect of outgroup size on (a) mean standard length (mm) and (b) mean dry weight (mg). Tank-triplet and group identity nested within tank-triplet were fitted as random intercepts (with variances shown). The reference level for Treatment was Control. Each table section displays the final model, with removed non-significant interactions below.

| **a. Offspring standard length (N=24 clutches)** | | | | | | |
| --- | --- | --- | --- | --- | --- | --- |
| Random terms: Tank-triplet: 0.00; Tank-triplet/Group: 1.33; Residual: 0.35 | | | | | | |
| FINAL MODEL | estimate ± s.e. | C.I. | df | t-value | p | *Χ*^2^ |
| Intercept | 12.17 ± 0.75 | 10.75 – 13.58 | 20.00 | 16.27 | <0.001 |  |
| Treatment |  |  | 1 |  | 0.028 | 4.85 |
| Treatment (Intruded) | -1.59 ± 0.71 | -2.96 – -0.21 | 9.53 | -2.23 | 0.052 |  |
| Treatment duration | -0.04 ± 0.01 | -0.05 – -0.02 | 11.41 | -4.78 | <0.001 |  |
| Number of living offspring | -0.01 ± 0.01 | -0.03 – 0.00 | 11.47 | -1.72 | 0.112 |  |
| REMOVED INTERACTION |  |  | df |  | p | *Χ*^2^ |
| Treatment x Treatment duration |  |  | 1 |  | 0.143 | 2.15 |
| **b. Offspring dry weight (N=24 clutches)** | | | | | | |
| Random terms: Tank-triplet: 1.99; Tank-triplet/Group: 0.25; Residual: 1.41 | | | | | | |
| FINAL MODEL | estimate ± s.e. | C.I. | df | t-value | p | *Χ*^2^ |
| Intercept | 6.89 ± 1.27 | 4.51 – 9.29 | 18.96 | 5.44 | <0.001 |  |
| Treatment |  |  | 1 |  | 0.089 | 2.89 |
| Treatment (Intruded) | -1.29 ± 0.73 | -2.75 – 0.31 | 3.88 | -1.77 | 0.154 |  |
| Treatment duration | -0.02 ± 0.02 | -0.05 – 0.01 | 13.51 | -1.55 | 0.145 |  |
| Number of living offspring | -0.02 ± 0.02 | -0.06 – 0.00 | 13.89 | -1.59 | 0.134 |  |
| REMOVED INTERACTION |  |  | df |  | p | *Χ*^2^ |
| Treatment x Treatment duration |  |  | 1 |  | 0.70 | 0.15 |
