# Supplementary material for: Experimental evidence that chronic outgroup conflict reduces reproductive success in a cooperatively breeding fish
Source: eLife. 2022 Sep 14;11:e72567. doi: 10.7554/eLife.72567 (PMC9473690; doi:10.7554/eLife.72567)
Supplement: Transparent reporting form [file elife-72567-transrepform1.pdf]

Westbrook Centre
Milton Road
Cambridge CB4 1YG, UK
P 01223 855340
W elifesciences.org
T @elife

eLife's transparent reporting form

We encourage authors to provide detailed information *within their submission* to facilitate the interpretation and replication of experiments. Authors can upload supporting documentation to indicate the use of appropriate reporting guidelines for health-related research (see EQUATOR Network), life science research (see the BioSharing Information Resource), or the ARRIVE guidelines for reporting work involving animal research. Where applicable, authors should refer to any relevant reporting standards documents in this form.

If you have any questions, please consult our Journal Policies and/or contact us: editorial@elifesciences.org.

Sample-size estimation

- You should state whether an appropriate sample size was computed when the study was being designed
- You should state the statistical method of sample size computation and any required assumptions
- If no explicit power analysis was used, you should describe how you decided what sample (replicate) size (number) to use

Please outline where this information can be found within the submission (e.g., sections or figure legends), or explain why this information doesn't apply to your submission:

Three factors played important roles in our decision to use 10 groups for each of our treatments (N = 10 Control groups, N = 10 Intruded groups): 1) given the long-term nature of our experiments, for ethical reasons, we wanted to minimise the number of individuals used whilst ensuring sufficient statistical power to detect treatment effects, 2) we used a matched-samples experimental design that requires smaller samples sizes than independent samples designs, and 3) previous laboratory experimental work on this species successfully detected treatment effects on reproductive measures N = 10 groups per treatment (Mileva et al. 2011).

Replicates

- You should report how often each experiment was performed
- You should include a definition of biological versus technical replication
- The data obtained should be provided and sufficient information should be provided to indicate the number of independent biological and/or technical replicates
- If you encountered any outliers, you should describe how these were handled
- Criteria for exclusion/inclusion of data should be clearly stated
- High-throughput sequence data should be uploaded before submission, with a private link for reviewers provided (these are available from both GEO and ArrayExpress)

Please outline where this information can be found within the submission (e.g., sections or figure legends), or explain why this information doesn't apply to your submission:

eLife Sciences Publications, Ltd is a limited liability non-profit non-stock corporation incorporated in the State of Delaware, USA, with company number 5030732, and is registered in the UK with company number FC030576 and branch number BR015634 at the address Westbrook Centre, Milton Road, Cambridge CB4 1YG.

Each experiment was conducted once.
Focal social groups were considered the technical replicate and the basis of biological replication, controlled for when multiple datapoints were collected from each group.
Description of all analyses is provided in the Methods, and number of datapoints is provided in figure legends and in Supplementary Information tables; all raw data used in the analyses described have been made available in Dryad.
All valid datapoints were included in the analyses. Clutches produced in the initial days of the experiments were excluded because they were deemed not to have had sufficient time to be affected by the treatments, and five clutches in Experiment I were excluded from analyses of hatching success because they failed to develop for reasons external to the treatments; this information is provided in the Methods.

eLife Sciences Publications, Ltd is a limited liability non-profit non-stock corporation incorporated in the State of Delaware, USA, with company number 5030732, and is registered in the UK with company number FC030576 and branch number BR015634 at the address Westbrook Centre, Milton Road, Cambridge CB4 1YG.

Statistical reporting

- Statistical analysis methods should be described and justified
- Raw data should be presented in figures whenever informative to do so (typically when N per group is less than 10)
- For each experiment, you should identify the statistical tests used, exact values of N, definitions of center, methods of multiple test correction, and dispersion and precision measures (e.g., mean, median, SD, SEM, confidence intervals; and, for the major substantive results, a measure of effect size (e.g., Pearson's r, Cohen's d)
- Report exact p-values wherever possible alongside the summary statistics and 95% confidence intervals. These should be reported for all key questions and not only when the p-value is less than 0.05.

Please outline where this information can be found within the submission (e.g., sections or figure legends), or explain why this information doesn't apply to your submission:

All statistical methods used are described in the Methods. Raw data are presented in all figures. Parameter estimates with associated confidence intervals and p-values are provided in the text and in Supplementary Information tables.

(For large datasets, or papers with a very large number of statistical tests, you may upload a single table file with tests, Ns, etc., with reference to sections in the manuscript.)

Group allocation

- Indicate how samples were allocated into experimental groups (in the case of clinical studies, please specify allocation to treatment method); if randomization was used, please also state if restricted randomization was applied
- Indicate if masking was used during group allocation, data collection and/or data analysis

Please outline where this information can be found within the submission (e.g., sections or figure legends), or explain why this information doesn't apply to your submission:

For each triplet, we randomly allocated (by flip of a coin) the side tanks to one of two experimental treatments, Control and Intruded.
Biological samples (eggs, offspring, protein and lipid extracts), offspring and parental-care videos were coded for blind processing. Intrusion videos could not be masked according to treatment (Intruded vs Control) but were masked with regards to time point in the experiment.

Additional data files ("source data")

- We encourage you to upload relevant additional data files, such as numerical data that are represented as a graph in a figure, or as a summary table
- Where provided, these should be in the most useful format, and they can be uploaded as "Source data" files linked to a main figure or table
- Include model definition files including the full list of parameters used
- Include code used for data analysis (e.g., R, MatLab)
- Avoid stating that data files are "available upon request"

Please indicate the figures or tables for which source data files have been provided:

Raw data have been made available in Dryad.

eLife Sciences Publications, Ltd is a limited liability non-profit non-stock corporation incorporated in the State of Delaware, USA, with company number 5030732, and is registered in the UK with company number FC030576 and branch number BR015634 at the address Westbrook Centre, Milton Road, Cambridge CB4 1YG.
